# Emerging therapeutic potential of glucagon-like Peptide-1 receptor agonists in knee osteoarthritis: a systematic review

**DOI:** 10.3389/fphar.2025.1627691

**Published:** 2025-10-13

**Authors:** Yapeng Li, Lanbo Yang, Feng Li, Jia Fu, Wangyu Zhao, Xiaolong Wu, Jiayi Guo, Chen Yue

**Affiliations:** ^1^ Rehabilitation Therapy Center, Luoyang Orthopedic Hospital of Henan Province, Luoyang, China; ^2^ Sports Medicine Center, Luoyang Orthopedic Hospital of Henan Province, Luoyang, China; ^3^ Non-Surgical Therapy Center for Osteoarthritis, Luoyang Orthopedic Hospital of Henan Province, Luoyang, China; ^4^ Department of Geratology, Xiyuan Hospital, China Academy of Chinese Medical Sciences, Beijing, China; ^5^ College of Orthopedics and Traumatology, Henan University of Traditional Chinese Medicine, Zhengzhou, China; ^6^ Department of Evidence-based Medicine, Luoyang Orthopedic Hospital of Henan Province, Luoyang, China

**Keywords:** knee osteoarthritis, glucagon-like peptide-1 receptor agonists, body weight, glycemic control, knee pain

## Abstract

**Objective:**

This study aims to systematically investigate the clinical efficacy and mechanisms of glucagon-like peptide-1 (GLP-1) receptor agonists (GLP-1 RAs) in the treatment of knee osteoarthritis (KOA), elucidate their underlying mechanisms, and propose potential future research directions.

**Design:**

This study followed the Preferred Reporting Items for Systematic Reviews and Meta-Analyses guidelines. We reviewed literature from PubMed, Embase, Web of Science, Cochrane Library, and ClinicalTrials.gov up to 31 December 2024. The search strategy combined “GLP-1″ and “KOA”. We included studies on GLP-1 RAs and KOA in humans and animals, excluding conference abstracts, reviews, letters, case reports, and other similar types of publications.

**Findings:**

Fifteen studies were included, covering six clinical investigations and nine fundamental research studies. Clinical evidence showed GLP-1 RAs significantly improved pain scores and function while reducing KOA incidence. Mechanistic studies reveal multi-target effects, including: 1) Metabolic regulation, 2) Anti-inflammatory action, and 3) Cartilage preservation through autophagy activation and apoptosis inhibition. Safety analysis notes gastrointestinal and tumor events. At the same time, we are concerned about a declining trend in long-term compliance with GLP-1 RAs.

**Conclusion:**

These findings positioned GLP-1 RAs as promising disease-modifying agents for metabolic-associated KOA, particularly in obese or diabetic subpopulations. While current evidence supports therapeutic potential, confirmatory phase III trials and long-term safety monitoring are needed to establish clinical guidelines.

**Systematic Review:**

https://www.crd.york.ac.uk/PROSPERO2/view/CRD420250656321, Identifier, CRD420250656321.

## Introduction

Knee osteoarthritis (KOA), a prevalent chronic degenerative joint disorder, is predominantly characterized by the progressive deterioration of articular cartilage. Its pathogenesis involves aging, obesity, joint injury, genetic predisposition, biomechanical imbalance, and lifestyle factors (Giorgino et al., 2023; Gelber, 2024). As the disease progresses, patients typically present with a constellation of symptoms, including persistent joint pain, stiffness, swelling, and progressive limitation of motion. A 2020 cohort study reported a global prevalence of KOA of 16.0% among individuals aged ≥15 years, increasing to 22.9% in those ≥40 years (Cui et al., 2020). Recent projections suggested a substantial 74.9% increase in KOA prevalence by 2050 compared to 2020 (Steinmetz et al., 2023). The disease burden of KOA is profound, with lifetime medical costs per patient in the United States reaching up to $140,300 and imposing significant economic burdens through productivity losses that affect individuals, families, and society (Losina et al., 2015; Leifer et al., 2022).

Recent research underscores the link between metabolic disorders, particularly obesity and diabetes, and KOA (Dubey et al., 2018; Eitner et al., 2021; Chowdhury et al., 2022; Wei et al., 2023). Obese individuals face a threefold higher KOA risk compared to healthy-weight individuals (Reyes et al., 2016). A cross-sectional study also found a positive correlation between dietary glycemic index and KOA prevalence in women (So et al., 2018). Intriguingly, overweight and obese individuals also show an increased risk of hand osteoarthritis, suggesting systemic effects beyond mechanical loading (Reyes et al., 2016; Plotz et al., 2021; Badley et al., 2022). Given the established link between metabolic dysregulation and KOA progression, pharmacological interventions targeting metabolic pathways have emerged as potential therapeutic candidates.

Glucagon-like peptide-1 (GLP-1) receptor agonists (GLP-1 RAs), a novel class of antidiabetic agents acting on GLP-1 receptors, exert dual glycemic control and weight-loss effects via enhancing insulin secretion, appetite suppression, and delayed gastric emptying (Ard et al., 2021). Emerging evidence suggests their potential extra-glycemic benefits, including anti-inflammatory and chondroprotective properties, may synergistically ameliorate KOA progression. The Phase 3 STEP9 trial showed that semaglutide, a kind of GLP-1 RAs, reduces body weight and alleviates knee pain in obese KOA patients (Bliddal et al., 2024). However, some studies, like a placebo-controlled trial, found no significant pain reduction with liraglutide (Gudbergsen et al., 2021). A case report also noted joint pain in a patient on liraglutide, which resolved after discontinuation (Ambrosio et al., 2014). Available evidence indicates that GLP-1 RAs are commonly associated with gastrointestinal adverse events, which may curtail long-term adherence (Bliddal et al., 2024; Gudbergsen et al., 2021).

Among GLP-1 RAs, semaglutide and tirzepatide are primarily indicated for weight management, while dulaglutide is predominantly used for glycemic control and liraglutide for both indications. However, since all GLP-1 RAs share a common mechanistic pathway through GLP-1 receptor activation—and given that this review focuses specifically on KOA outcomes—we will analyze them collectively as a single pharmacological class rather than distinguishing between individual agents. Currently, there remains a critical gap in the literature regarding the comprehensive effects of GLP-1 RAs on KOA. This study, therefore, aims to (1) systematically evaluate the clinical efficacy of GLP-1 RAs in KOA management, (2) elucidate their underlying therapeutic mechanisms, and (3) identify key directions for future research in this emerging field.

## Methods

This systematic review was registered on the PROSPERO (Registration number: CRD420250656321). This study followed the Preferred Reporting Items for Systematic Reviews and Meta-Analyses (PRISMA) guidelines. (Appendix S1).

## Literature search

We performed a comprehensive search across PubMed, Embase, Web of Science, Cochrane Library, and ClinicalTrials.gov up to 31 December 2024. Reference lists of relevant reviews were also screened. The search strategy combined MeSH terms and free-text words, including “Glucagon-Like Peptide 1 [MeSH]”, “GLP-1″, “Semaglutide”, “Liraglutide”, “Saxenda”, “Tirzepatide”, “Albiglutide”, “Exenatide”, “Dulaglutide”, “Beinaglutide”, “Polyethylene glycol loxenatide”, “Lixisenatide”, “Loxenatide”, “Mashidutide”, paired with “Osteoarthritis, knee [MeSH]”, “Degeneration of the knee”, “Knee joint”, “Knee OA”, and “KOA”. The full search strategy is shown in Appendix S2.

### Inclusion and exclusion criteria

Eligible studies included original research investigating GLP-1 RAs in KOA, using either human participants or animal models, which reported outcomes involving pain, function, safety, cartilage degradation, or mechanistic pathways. Studies were excluded if they constituted reviews, case reports, conference abstracts, or letters.

## Literature screening and data extraction

Two researchers independently screened and extracted data, with a third resolving discrepancies. The process involved (1) searching databases and removing duplicates; (2) excluding irrelevant works (e.g., conference papers, reviews, case reports, etc.); and (3) a full-text reading of remaining articles against inclusion criteria. Data extracted included study details, clinical trial characteristics, primary endpoints, and biochemical results.

### Quality assessment

The quality of included randomized controlled trials (RCTs) was assessed using the Cochrane tool “Risk of Bias 2”. For cohort studies, quality evaluation was performed using the Newcastle-Ottawa Scale (NOS) with a maximum score of 9 stars, where studies scoring <7 stars were classified as moderate/low quality and those with ≥7 stars as high quality (Cook and Reed, 2015). Non-comparative studies were appraised through the first 8 items of the Methodological Index for Non-Randomized Studies (MINORS), yielding a total score of 16 points. Quality stratification was defined as follows: 0–4 points (very low quality), 5-7 points (low quality), 8–12 points (moderate quality), and ≥13 points (high quality) (Slim et al., 2003). Given the exploratory nature of preclinical findings and heterogeneity in experimental designs, quality assessment was intentionally restricted to clinical evidence supporting primary outcomes.

## Results

**FIGURE 1:**
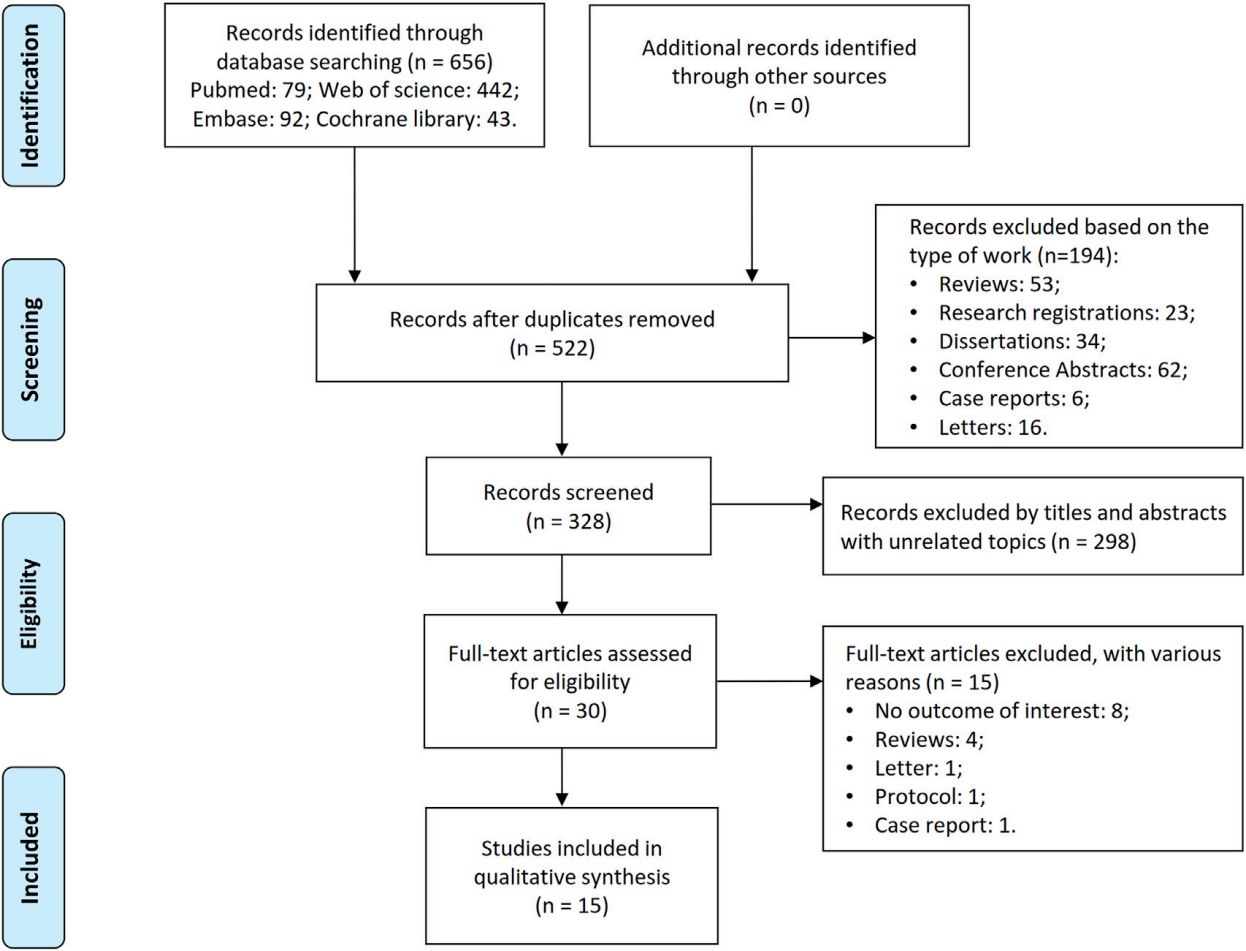
Flowchart of literature search and screening process.

The initial literature search identified 656 potentially relevant studies dedicated to 522 unique records. After excluding 194 non-targeted publications (e.g., conference proceedings, reviews, letters, case reports, research registries, etc.), 328 articles remained. Screening of titles and abstracts yielded 30 articles for full-text evaluation, with 15 meeting the inclusion criteria. The selection process is summarized in Figure 1.

## Characteristics of included studies

Fifteen studies included six clinical investigations and nine fundamental research studies.

### Clinical investigations

Three RCTs, two prospective cohort studies, and one single-arm interventional study were included. Participants were individuals with KOA who were obese or had diabetes, as well as obese individuals without KOA. Interventions involved GLP-1 RAs such as tirzepatide, semaglutide, liraglutide, etc. Treatment durations ranged from 6 months to 5 years, with some studies lacking specific timeframes. Despite the potential overlap in research teams, studies were included based on distinct data collection timelines and methodological differences (Bliddal et al., 2024; Gudbergsen et al., 2021; Bartholdy et al., 2022; Table 1).

**TABLE 1:** Basic characteristics of the included clinical trials.

Study	Country	Study design	Population	Age (mean ± SD) Experimental/Control	Sample size (F) Experimental/Control	Medical prescription		Duration of use of drugs	Follow-up time	Outcome indicators	NOS/MINORS
						Experimental	Control				
Bartholdy et al. (2022)	Denmark	Randomized controlled trial	KOA with K-L graded from 1 to 3 Age 18 to 74 Overweight or obesity (BMI≥27 kg/m^2^), with or without T2D (5.8%–10.6%) Weight loss ≥5% after the 8-week intensive dietary intervention	58.8 ± 11.3/58.6 ± 9.6	66(43)/69(44)	Liraglutide: Starting with 0.6 mg/day increasing biweekly by 0.6 mg/day until 3 mg/day	Placebo: Identically appearing placebo	1 year	1 year	Physical activity KOOS function Body weight	
Bliddal et al. (2024)	Denmark	Double-blind, randomized, placebo-controlled trial	KOA with K-L graded 2 or 3 Age ≥18 Obesity with BMI ≥30 kg/m^2^ WOMAC pain score (0–100, the higher the score, the worse the pain.) ≥ 40	56.0 ± 10.0/56.0 ± 10.0	271(228)/136(104)	Semaglutide: Once-weekly subcutaneous semaglutide (initiated at a dose of 0.24 mg, with dose escalation intended to reach the 2.4-mg target at week 16)	Placebo: Visually identical placebo	68 weeks	7 weeks	Body weight WOMAC SF-36	
Gudbergsen et al. (2021)	Denmark	Randomized controlled trial	KOA with K-L graded from 1 to 3 Age 18 to 74 Overweight or obesity with BMI≥27 kg/m^2^ Weight loss ≥5% after the 8-week intensive dietary intervention	59.2 ± 10.8/59.3 ± 9.7	80(52)/76(49)	Liraglutide: Starting with 0.6 mg/d and followed by incremental biweekly dose escalation steps of 0.6 mg/d to liraglutide 3 mg/d	Placebo: Identically appearing placebo	52 weeks	Once at 4-week intervals during treatment	Body weight KOOS pain subscale ICOAP questionnaire KOOS score WOMAC Anthropometry Responder indices	
Lavu et al. (2024)	United States of America	Retrospective cohort study	Obese diabetic with BMI ≥30 kg/m^2^ Had a T2D diagnosis Followed up for at least 5 years Pre-existing hip and/or knee OA was excluded	55.4 ± 11.7/55.4 ± 12.4	15693(9037)/15693(9003)	GLP-1 RAs	No GLP-1 RAs	Patients initiated during their initial visit between 2015 and 2017 and were followed until the conclusion of the study in 2020–2022	At least 5 years	Rates of diagnosis for KOA/TKA BMI HbA1c	8
			Obese non-diabetic with BMI ≥30 kg/m^2^ Had no a T2D diagnosis Followed up for at least 5 years Pre-existing hip and/or knee OA was excluded	47.4 ± 12.9/47.4 ± 12.9	1859(1502)/1859(1507)	GLP-1 RAs	No GLP-1 RAs	Patients initiated during their initial visit between 2015 and 2017 and were followed until the conclusion of the study in 2020–2022	At least 5 years	Rates of diagnosis for KOA/TKA BMI HbA1c	
			Non-obese diabetic patients with BMI ≤30 kg/m^2^ Had a T2D diagnosis Followed up for at least 5 years Pre-existing hip and/or knee OA was excluded	58.3 ± 11.5/58.0 ± 12.2	6019(3382)/6019(3320)	GLP-1 RAs	No GLP-1 RAs	Patients initiated during their initial visit between 2015 and 2017 and were followed until the conclusion of the study in 2020–2022	At least 5 years	Rates of diagnosis for KOA/TKA BMI HbA1c	
Zhu et al. (2023)	China	Prospective, observational, multicentre cohort study	KOA with T2D K-L graded from 1 to 3 Followed up for at least 5 years	60.7 ± 8.7/61.2 ± 8.6	233(174)/1574(1145)	Among GLP-1 RA users, 93.5% were concurrently prescribed oral antidiabetic drugs and 63.5% received concomitant insulin therapy	Among Non-GLP-1 RAs users, 92.4% were concurrently prescribed oral antidiabetic drugs and 63.0% received concomitant insulin therapy	At least 2 years, average about 4.9 years	At least 5 years, average 7.7–7.8 years	Knee surgery incidence Pain-relieving medication use Number of intra-articular Therapies WOMAC Cartilage thickness Medial femorotibial joint cartilage thickness	9
Samajdar et al. (2024)	India	Single-arm, record-based observational study	KOA with T2D Age ≥60 years Conventional treatment for at least 3 months	67.1 ± 4.5	98 (40)	Patients received dulaglutide added to ongoing anti-diabetes regimens; existing medications like sitagliptin, linagliptin and vildagliptin were stopped. Concomitant OA treatments (including NSAIDs) were used for at least 3 months prior		At least 6 months	Baseline, after 3 months and 6 months	Glycemic metrics VAS scores NSAIDs consumption Body weight BMI.	7

BMI, body mass index; F, female; GLP-1 RAs, Glucagon-like peptide-1 receptor agonists; HbA1c, Hemoglobin A1c; ICOAP, intermittent and constant osteoarthritis pain; K-L, Kellgren-Lawrence score; KOA, knee osteoarthritis; KOOS, knee injury and osteoarthritis outcome score; MINORS, Methodological Index for Non-randomized Studies; NOS, Newcastle-Ottawa scale; NSAIDs, Non-steroidal anti-inflammatory drugs; SD, standard deviation; SF-36, 36-Item Short Form Health Survey; T2D, Type 2 diabetes; TKA, total knee arthroplasties; VAS, visual analogue scale; WOMAC, Western Ontario and McMaster Universities Osteoarthritis Index.

### Fundamental research

Focused on animal models and cellular experiments. Animal studies assessed body weight, functional scores, pain behavior, and histomorphological changes. Cellular experiments explored inflammation, oxidative stress (OS), matrix metabolism, apoptosis, autophagy, and signaling pathways.

### Quality assessment

**FIGURE 2:**
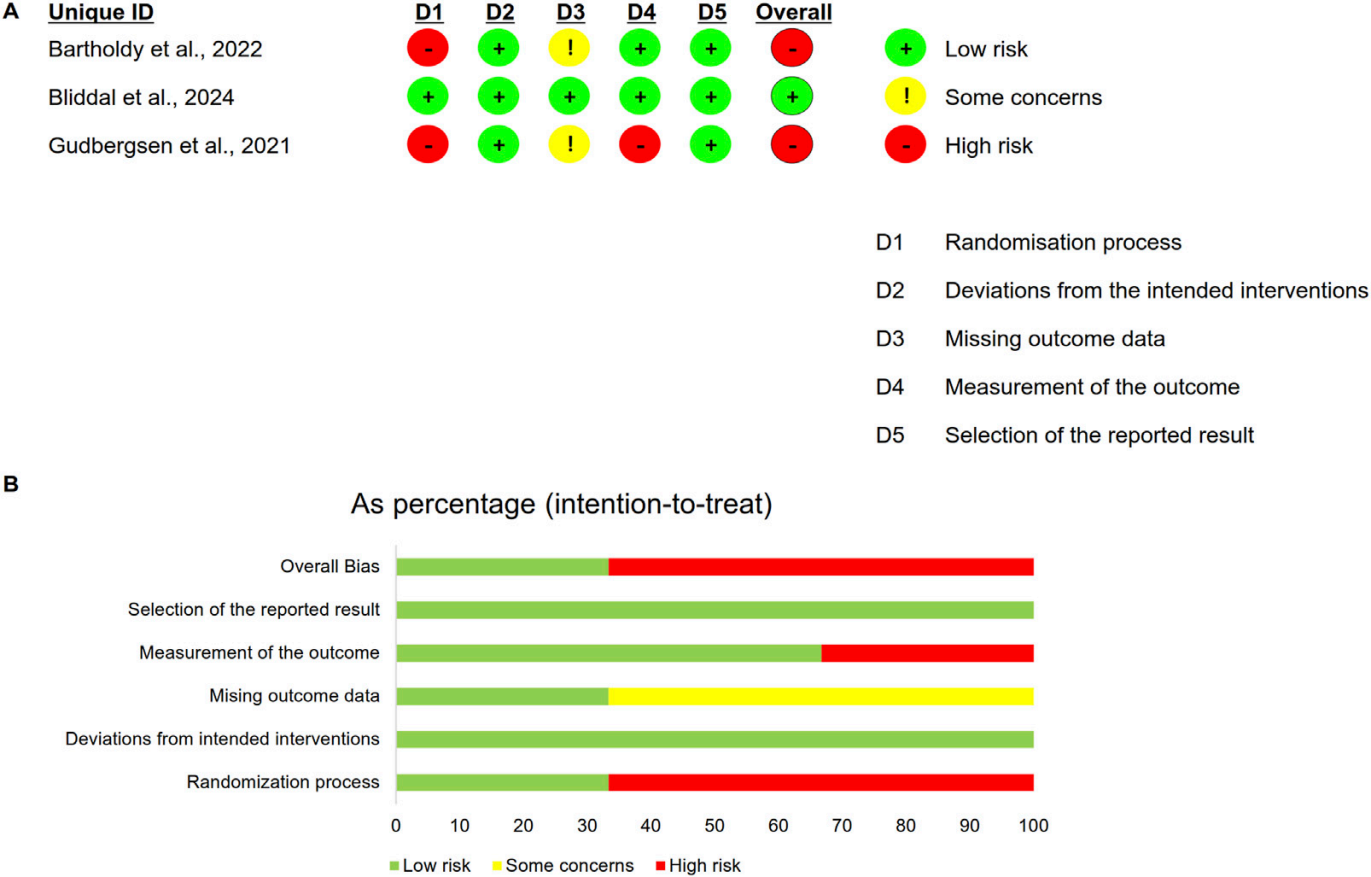
Risk of bias for RCTs. **(A)** Summary of risk of bias; **(B)** Percentage of risk of bias.

Two RCTs were rated as high risk of bias due to inadequate reporting of randomization procedures (Figure 2). Both cohort studies achieved NOS scores exceeding 7 stars, meeting predefined thresholds for high methodological quality. The single-arm study received a low-quality rating (MINORS score: 7/16) primarily attributable to its non-prospective design and substantial loss to follow-up (12.5%) (Table 1).

## Clinical efficacy of GLP-1 RAs on KOA

The primary outcomes, which include weight, pain, joint function, KOA risk, and safety, are summarized in Table 2.

**TABLE 2:** Clinical trial outcomes of GLP-1 RAs in the treatment of KOA.

Study	Body weight	Pain	Function	Risk of KOA	Other	Safety
Bartholdy et al. (2022)	+		+			
Bliddal et al. (2024)	+	+	+			-
Gudbergsen et al. (2021)	+	=	=			-
Lavu et al. (2024)	=			-		
Samajdar et al. (2024)	+	=			Blood glucose (+)	
Zhu et al. (2023)	+	+	+	+	Cartilage (+)	

The symbol “+” indicates a favoring of the GLP-1 RAs group; the symbol “ = “ indicates that the two groups are equivalent; and the symbol “-“ indicates a favoring of the control group.

GLP-1 RAs, Glucagon-like peptide-1 receptor agonists; KOA, knee osteoarthritis.

### Body weight

Six studies evaluated body weight changes. One RCT (Gudbergsen et al., 2021) reported a 2.8 kg weight loss with liraglutide at 52 weeks versus a 1.2 kg gain in the placebo group (P = 0.008). A significantly higher proportion of patients in the liraglutide group achieved >5% weight loss compared to the placebo group (35% vs. 17.1%, P = 0.024), although no difference reached statistical significance for the more stringent >10% weight loss threshold. Another RCT (Bartholdy et al., 2022) found that liraglutide produced significantly greater weight loss than placebo in participants with overweight or obesity (mean between-group difference 4.1 kg, 95% CI −6.0 kg to −2.1 kg; P < 0.0001). Similarly, semaglutide led to a 13.7% weight loss at 68 weeks, compared to 3.2% in the placebo group (P < 0.001) (Bliddal et al., 2024). A cohort study (Zhu et al., 2023) of KOA patients with type 2 diabetes (T2D) reported significantly greater weight loss in the GLP-1 RAs group (mean difference −7.29 kg; 95% CI −8.07 to −6.50 kg; P < 0.001), although 42.06% of GLP-1 RAs users still maintained or gained weight. In the single-arm study by Samajdar et al. (2024) of 40 patients with KOA and T2D, dulaglutide reduced mean body weight from 81.6 ± 8.4 kg to 73.3 ± 7.5 kg (−8.3 kg, P < 0.001) and mean body mass index (BMI) from 30.6 ± 3.5 kg/m^2^ to 27.5 ± 3.2 kg/m^2^ (−3.1 kg/m^2^, P < 0.001) over 6 months. However, an investigation (Lavu et al., 2024) found no statistically significant difference in BMI changes over 2 years between patients receiving GLP-1 RAs and those who did not.

### Pain

Four studies assessed pain. One RCT (Gudbergsen et al., 2021) found no significant differences between the groups in pain trajectory or the Western Ontario and McMaster Universities Osteoarthritis Index (WOMAC) pain subscale scores over 52 weeks. Another RCT (Bliddal et al., 2024) reported that semaglutide significantly reduced the WOMAC pain subscale scores (41.7 vs. 27.5 points, P < 0.001) and the use of non-steroidal anti-inflammatory drugs (NSAIDs) or acetaminophen. A cohort study (Zhu et al., 2023) reported significant WOMAC pain subscale score improvements in the GLP-1 RAs group, independent of weight loss or Hemoglobin A1c (HbA1c). Compared to the non-GLP-1 RAs group, the GLP-1 RAs group showed only numerical, non-statistically significant reductions in the annual consumption of oral NSAIDs, acetaminophen, topical NSAIDs, opioids, and the number of intra-articular treatments. The frequency of intra-articular steroid injections was markedly diminished in the GLP-1 RAs group, with an adjusted mean difference of −0.087 injections per year (95% CI −0.14 to −0.036; P < 0.001). Within-group, the GLP-1 RAs group revealed significant improvements in all pain-related metrics except for opioid use. Similarly, a single-arm study (Samajdar et al., 2024) demonstrated that dulaglutide improved pain and reduced NSAIDs consumption.

### Function

Four studies evaluated functional outcomes. Liraglutide (Bartholdy et al., 2022) improved the Knee Injury and Osteoarthritis Outcome Score (KOOS) function scores by 3.7 points (baseline 81.0 ± 15.1) versus −0.1 for placebo (baseline 85.1 ± 10.7); between-group difference 3.8 points (95% CI 0.9–6.7, P = 0.01). Semaglutide (Bliddal et al., 2024) reduced WOMAC physical function by 14.9 points (95% CI −20.4 to −9.3, P < 0.001) and stiffness by 15.9 points (95% CI −23.2 to −8.6, P < 0.001), and increased 6-min walk distance by 42.6 m versus placebo (95% CI 25.6–59.7, P < 0.001). However, one RCT (Gudbergsen et al., 2021) found no significant differences in KOOS subscales. A cohort study (Zhu et al., 2023) found the GLP-1 RA group achieved significantly greater WOMAC total score improvement than controls (adjusted mean difference −1.46, 95% CI −2.84 to −0.08; P = 0.038).

### KOA risk

Two cohort studies examined KOA risk. One (Zhu et al., 2023) found a lower surgery rate in the GLP-1 RAs group (1.7% vs. 5.9%, P = 0.005), while another (Lavu et al., 2024) reported a higher KOA prevalence in GLP-1 RAs users (11.0% vs. 7.4%, P < 0.05).

## Other outcomes

**Cartilage Degeneration:** A cohort study (Zhu et al., 2023) found slower cartilage degeneration in GLP-1 RA users (P = 0.026), independent of weight loss or HbA1c.

**Glycemic Control:** A single-arm study (Samajdar et al., 2024) reported dulaglutide improved HbA1c, fasting glucose, and postprandial glucose, with pain reduction correlating with HbA1c improvements.

**Blood Pressure:** Semaglutide reduced systolic and diastolic blood pressure (Bliddal et al., 2024).

**Treatment Adherence:** A cohort study (Lavu et al., 2024) noted declining GLP-1 RAs utilization over time.

### Safety

Three studies reported adverse events. Liraglutide had a higher withdrawal rate (12.5% vs. 5.3%), primarily due to gastrointestinal disorders (Gudbergsen et al., 2021). Semaglutide showed a slightly higher adverse event rate (10.0% vs. 8.1%), with tumor-related and gastrointestinal events being the most common (Bliddal et al., 2024). Dulaglutide was generally well-tolerated, with manageable gastrointestinal side effects (Samajdar et al., 2024).

## Fundamental research on the effects of GLP-1 RAs on KOA

### GLP-1 receptor expression

Multiple studies have reported GLP-1 receptor expression in articular cartilage, synovium, and other joint tissues. Compared with healthy cartilage, degenerated cartilage shows lower GLP-1 receptor expression (Chen et al., 2018); similar downregulation is observed in monosodium iodoacetate (MIA) -treated rat cartilage (Que et al., 2019). In human chondrocytes, advanced glycation end products (AGEs) further reduce GLP-1 receptor levels. GLP-1 receptors are distributed across cartilage, meniscus, bone marrow, and synovial tissue in both KOA and healthy models (Meurot et al., 2022). Liraglutide treatment increases GLP-1 receptor expression in degenerative models (Que et al., 2019).

### Analgesic effects

Meurot et al. (2022) showed that the GLP-1 RA liraglutide produces robust, dose-dependent analgesia in a murine model of osteoarthritis. A single intra-articular injection elevated paw-withdrawal thresholds within 3 days, and the benefit persisted through day 10. At 20 μg, liraglutide matched 20 µg dexamethasone on days 3 and 7 and surpassed it on day 10, while simultaneously attenuating synovitis. In a 28-day regimen, liraglutide sustained analgesia beyond the corticosteroid’s waning effect, outperforming dexamethasone on day 14 and matching it on days 21 and 28.

#### Body weight

Two studies investigated the effects of GLP-1 RAs on body weight in murine models of osteoarthritis, yet reported contradictory findings. The first study (Que et al., 2019) demonstrated a significant reduction in body weight in osteoarthritis model rats following liraglutide administration. In contrast, the second study (Meurot et al., 2022) revealed that neither short-term (10-day) nor extended (28-day) liraglutide treatment exerted any measurable effect on body weight in osteoarthritis model mice.

#### Anti-inflammatory

Seven studies have demonstrated the anti-inflammatory effects of GLP-1 RAs. Liraglutide (Que et al., 2019) and lixisenatide (Li et al., 2019) both suppress tumor necrosis factor-α (TNF-α), interleukin (IL) −6, and IL-1β. Consistently, exenatide-4 (Tong et al., 2019) and liraglutide (Mei et al., 2019) concurrently attenuate gene and protein expression of TNF-α, IL-1β, IL-6, and monocyte chemoattractant protein (MCP) −1. Dulaglutide further elevates prostaglandin E2 (PGE2) and its synthesizing enzyme cyclooxygenase 2 (COX-2) while repressing IL-6, IL-8, and MCP-1 in human SW1353 chondrocytes at both transcriptional and translational levels (Li et al., 2020). In murine primary chondrocytes, liraglutide dose-dependently diminishes secretion of nitrite, PGE2, and IL-6, and similar concentration-dependent reductions in nitric oxide (NO), PGE2, and IL-6 release—together with decreased expression of IL-6, COX-2, and TNF-α—are observed in RAW264.7 macrophages. Mechanistically, liraglutide skews macrophage polarization toward an anti-inflammatory phenotype by down-regulating M1-associated MCP-1 and CD38 while up-regulating the M2 marker early growth response protein 2 (Meurot et al., 2022). Finally, one study (Zhang et al., 2024) showed that liraglutide blunts AGEs–induced production of IL-1β, IL-6, IL-12, and TNF-α in primary chondrocytes, underscoring its broad anti-inflammatory potential.

### Oxidative stress

Four studies demonstrated that GLP-1 RAs reduce OS. One study (Tong et al., 2019) found that exendin-4 dose-dependently lowered reactive oxygen species (ROS) production and reversed AGEs-induced glutathione depletion. Similarly, another study (Li et al., 2019) showed that lixisenatide (20 nM) normalized OS markers 4-hydroxynonenal (4-HNE) and NADPH oxidase 4 (NOX-4) to near-basal levels. One study (Mei et al., 2019) reported that liraglutide dose-dependently suppressed TNF-α-induced ROS in osteoarthritis. Additionally, one study (Li et al., 2020) observed that dulaglutide (50 and 100 nM) significantly reduced AGEs-induced OS from 4.2-fold to 2.5-fold and 1.7-fold.

### Anti-catabolic

Eight studies demonstrated the anti-catabolic effects of GLP-1 RAs. Four studies showed that liraglutide downregulates the expression of matrix metalloproteinase-1/3/13 (MMP-1/3/13) and A disintegrin and metalloproteinase with thrombospondin motifs-4/5 (ADAMTS-4/5) while reducing glycosaminoglycan release from the cartilage extracellular matrix (ECM) (Chen et al., 2018; Meurot et al., 2022; Mei et al., 2019; Zhang et al., 2024), preserving type II collagen and aggrecan. Similar effects were observed with lixisenatide, exenatide, and dulaglutide, which also suppressed MMP-3/13 and ADAMTS-4/5 (Li et al., 2019; Tong et al., 2019; Li et al., 2020). Additionally, geniposide protected against MIA-induced osteoarthritis in rats by reducing MMP-13 and enhancing type II collagen expression (Huang et al., 2023).

#### Apoptosis

Two studies explored the role of GLP-1 RAs in apoptosis. One study (Chen et al., 2018) demonstrated that liraglutide reduced pro-apoptotic proteins (cleaved-caspase 3, Bax) while increasing anti-apoptotic Bcl-2 in chondrocytes. In rats, it decreased C/EBP homologous protein (CHOP) and caspase-3, alleviating osteoarthritis. A recent study (Zhang et al., 2024) further showed that liraglutide (>100 nM) attenuated AGE-induced chondrocyte apoptosis by suppressing caspase-3 and downregulating the receptor for advanced glycation end products (RAGE).

#### Autophagy

One study (Huang et al., 2023) demonstrated that GLP-1 RAs enhance autophagy in human normal chondrocyte C28/I2 cells by decreasing p62 and increasing Beclin-1 and LC3-II expression, thereby protecting chondrocytes.

### Mitochondrial dysfunction

A study (Li et al., 2019) demonstrated that lixisenatide dose-dependently restored AGE-induced reductions in mitochondrial membrane potential (MMP) and adenosine triphosphate (ATP), with 20 mg nearly normalizing both parameters.

#### Signaling pathway

Seven studies indicate that GLP-1 RAs protect articular cartilage by modulating Nuclear Factor Kappa-B (NF-κB), Protein Kinase A (PKA)/cyclic adenosine monophosphate response element-binding protein (CREB), Phosphoinositide 3-Kinase (PI3K)/Protein Kinase B (Akt), and AMP-activated Protein Kinase (AMPK)/Mammalian Target of Rapamycin (mTOR) pathways.

#### NF-κB pathway

The NF-κB pathway, linked to inflammation, apoptosis, and matrix degradation, is inhibited by liraglutide (Chen et al., 2018; Mei et al., 2019). Lixisenatide reduces AGE-induced IkBα phosphorylation, p65 nuclear translocation, and NF-κB activation (Li et al., 2019). Exendin-4 suppresses NF-κB activation by decreasing p38 phosphorylation, p65 nuclear translocation, and luciferase activity dose-dependently (Tong et al., 2019). Dulaglutide inhibits NF-κB activation by reducing AGE-mediated p65 nuclear translocation and luciferase activity in chondrocytes dose-dependently (Li et al., 2020).

#### PKA/CREB pathway

A study (Que et al., 2019) demonstrated that liraglutide activates the PKA/CREB pathway by upregulating PKA/p-PKA/CREB/p-CREB protein expression, contributing to its anti-inflammatory effects.

#### PI3K/Akt pathway

One study (Chen et al., 2018) showed that liraglutide inhibits endoplasmic reticulum (ER) stress through activation of PI3K/Akt signaling, which in turn reduces apoptotic protein activity and exerts a protective effect on cartilage.

#### AMPK/mTOR pathway

A recent study (Huang et al., 2023) demonstrated that geniposide dose-dependently upregulated GLP-1 receptor expression and protected articular cartilage through AMPK/mTOR-mediated autophagy (inhibiting mTOR while activating AMPK).

## Discussion

A notable strength of this review lies in its integration of diverse mechanistic evidence, spanning anti-inflammatory, anti-catabolic, and metabolic pathways. By synthesizing preclinical and clinical data, we elucidate how GLP-1 RAs may modulate key pathological processes in KOA, such as NF-κB inhibition, autophagy activation, and macrophage polarization. These insights not only support their therapeutic potential but also identify actionable targets for future research. Our findings demonstrated that GLP-1 RAs exerted significant therapeutic benefits, including pain alleviation, functional improvement, and reduced risk of KOA. These effects are mediated through multiple mechanisms, such as weight loss, anti-inflammatory, anti-catabolic, anti-apoptotic, regulation of ROS, and autophagy, demonstrating a comprehensive protective effect on KOA (Table 3). However, safety concerns have been identified regarding the clinical application of these medications, particularly their potential association with the complications of gastrointestinal and tumors, which warrant further investigation.

**TABLE 3:** The mechanism of action of GLP-1 RAs on KOA.

Study	GLP-1 RAs	Anti-catabolic	Anti-inflammatory	Oxidative stress	Apoptosis	Autophagy	Pathway
Chen et al. (2018)	Liraglutide	Type II collagen (+) MMP-3 (−)		ER stress (−)	CHOP, Caspase-3, Bax (−) Bcl-2 (+)		NF-Kb (−); PI3K/Akt (+)
Huang et al. (2023)	Geniposide	Type II collagen (+) MMP-13 (−)				p62 (−) Beclin-1 and LC 3-II (+)	AMPK (+)/mTOR (−)
Li H et al. (2020)	Dulaglutide	MMP-3/13 and ADAMTS-4/5 (−) Type II collagen and aggrecan (+)	PGE2(COX-2) (−) IL-6, IL-8, MCP-1 (−)	ROS (−)			NF-κB (−)
Li X et al. (2019)	Lixisenatide	MMP-3/13 and ADAMTS-4/5 (−) Type II collagen and aggrecan (+)	TNF-α, IL-6 (−)	4-HNE and NOX-4 (−) Mitochondria: MMP and ATP (+)			NF-κB (−)
Mei et al. (2019)	Liraglutide	MMP-3/13 and ADAMTS-4/5 (−) Type II collagen and aggrecan (+)	IL-6 and MCP-1 (−)	ROS and NOX-4 (−)			NF-κB (−)
Meurot et al. (2022)	Liraglutide	MMP-3/13 and ADAMTS-4/5 (−); Glycosaminoglycan (−)	Nitrite, PGE2, IL-6, NO (−) M1 to M2 macrophage shift				
Que et al. (2019)	Liraglutide		TNF-α, IL-6 and IL-1β (−)				PKA/CREB (+)
Tong et al. (2019)	Exenatide	MMP-3/13 and ADAMTS-4/5 (−) Type II collagen and aggrecan (+)	TNF-α and IL-1β (−)	ROS (−) Glutathione (+)			NF-κB (−)
Zhang et al. (2024)	Liraglutide	MMP-1/3/13 and ADAMTS-4/5 (−)	IL-1β, IL-6, IL-12 and TNF-α (−)		Caspase-3 and RAGE (−)		

4-HNE, 4-hydroxynonenal; ADAMTS, 4/5, A disintegrin and metalloproteinase with thrombospondin motifs 4/5; Akt, Protein Kinase B; AMPK, AMP-activated Protein Kinase; ATP, adenosine triphosphate; CHOP, C/EBP, homologous protein; COX-2, Cyclooxygenase 2; CREB, Cyclic adenosine monophosphate response element-binding protein; ER, endoplasmic reticulum; GLP-1 RAs, Glucagon-like peptide-1 receptor agonists; IL, interleukin; KOA, knee osteoarthritis; MCP, monocyte chemoattractant protein; MMP 1/3/13, Matrix metalloproteinase 1/3/13; MMP, mitochondrial membrane potential; mTOR, mammalian target of rapamycin; NF-κB, Nuclear Factor Kappa-B; NO, nitric oxide; NOX-4, NADPH, oxidase 4; PGE2, Prostaglandin E2; PI3K, Phosphoinositide 3-Kinase; PKA, Protein Kinase A; RAGE, receptor for advanced glycation end products; ROS, reactive oxygen species; TNF-α, tumor necrosis factor alpha.

Numerous studies have established a link between obesity, diabetes, and KOA progression. A study (Chen et al., 2020) demonstrated that overweight and obesity increase knee joint loading, which further exacerbates cartilage damage and joint deformity, thereby inducing and accelerating the onset and development of KOA. A meta-analysis (Williams et al., 2016) adjusted for BMI revealed a significant association between T2D and osteoarthritis symptoms. Data from the Osteoarthritis Initiative further showed that diabetes worsens KOA severity and impairs physical and mental health (Eitner et al., 2021). A further analysis (Alenazi et al., 2020) revealed that patients with poorer glycemic control exhibited heightened pain severity compared to those with better-controlled HbA1c levels. Conversely, a mediterranean diet with a lower glycemic index was associated with reduced KOA risk (Veronese et al., 2017). The evidence from these studies suggests that weight loss and glycemic control are crucial factors in KOA management in patients with obesity and/or diabetes.

Obesity or diabetes contributes to KOA development through multiple mechanisms. Adipose tissue in obese individuals secretes adipokines (e.g., leptin, lipocalin, resistin) and inflammatory factors (e.g., TNF-α, IL-1, IL-6), impacting KOA progression (Chang et al., 2018; Urban and Little, 2018). Leptin, for instance, promotes cartilage degradation by activating the NF-κB pathway and increasing inflammatory factors and MMP-1/13 (Abella et al., 2017). Diabetes affects KOA via chronic hyperglycemia, proinflammatory cytokines, OS, and insulin resistance (Wei et al., 2023). High glucose levels induce AGEs, which bind to chondrocyte RAGE receptors, activate NF-κB and mitogen-activated protein kinase (MAPK) pathways, and promote inflammatory factor release (e.g., IL-6, IL-8) and MMP-13 expression, exacerbating inflammation and ECM degradation (Rasheed et al., 2011). Hyperglycemia and adipose tissue create a low-grade inflammatory state, increasing pro-inflammatory factors (e.g., TNF-α, IL-1β, IL-6) and ECM degradation (Rogero and Calder, 2018; Wang and He, 2018). Obesity and diabetes elevate ROS levels, creating a pro-inflammatory environment that increases M1-type macrophages and cytokines, worsening OS and mitochondrial dysfunction (Niemann et al., 2017; Ahmed et al., 2021). ROS overproduction activates MAPK and NF-κB pathways, disrupting cartilage balance (Lepetsos et al., 2019; Rendra et al., 2019). Insulin resistance notably impacts KOA more severely in T2D patients (Eymard et al., 2015). Diabetes leads to severe synovial inflammation and elevated TNF-α levels in obese KOA patients, upregulating pro-inflammatory factors and MMP-13, and damaging joints (Hamada et al., 2016; Qiao et al., 2020). Additionally, leukocyte cell-derived chemotaxin 2, a metabolic factor primarily expressed in the liver, may influence glucose metabolism and obesity-related insulin resistance, potentially advancing the pathogenesis of KOA (Zhu et al., 2022).

**FIGURE 3:**
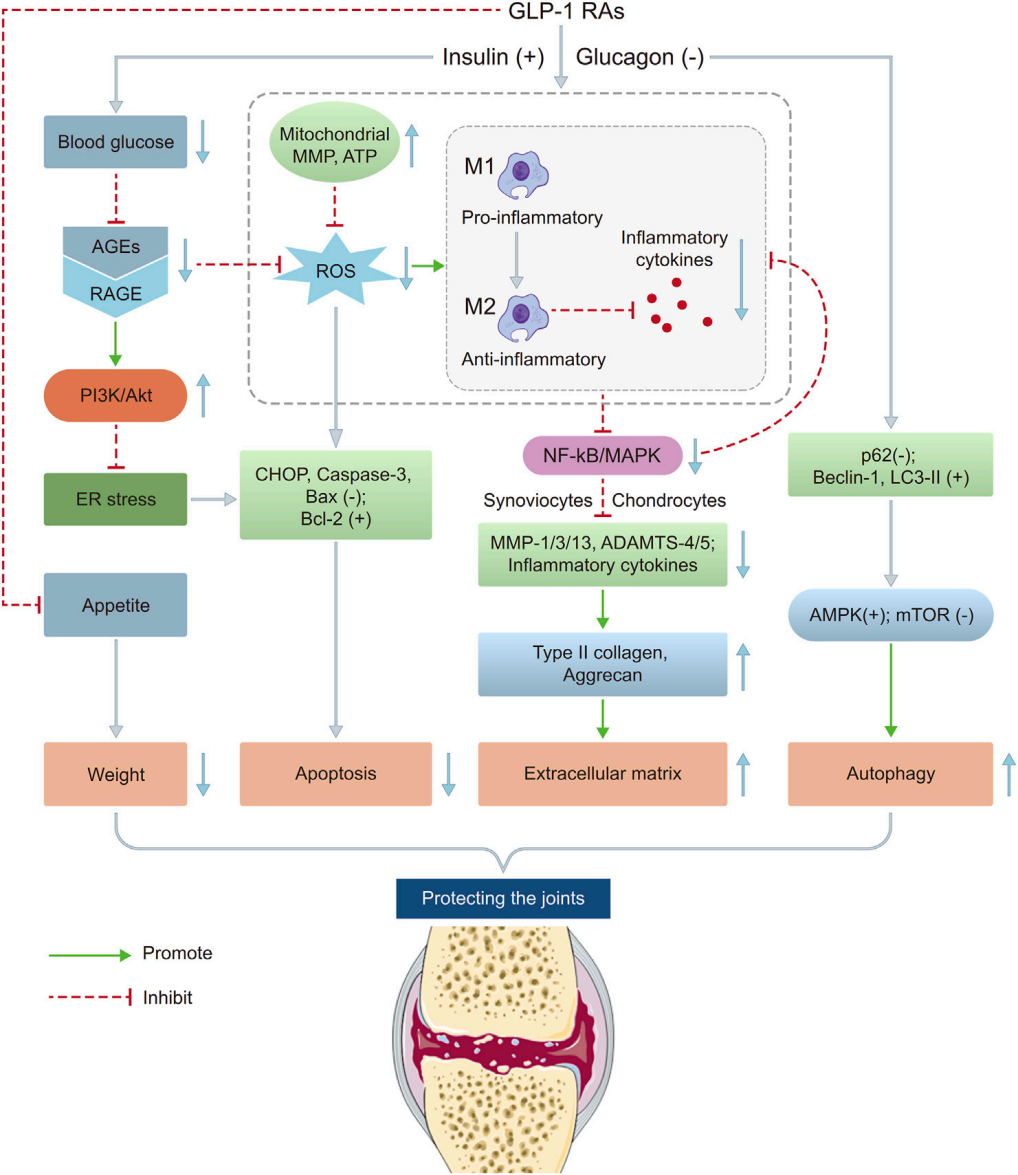
Potential mechanisms of GLP-1 RAs on KOA. This figure illustrates the potential mechanisms by which GLP-1 RAs may influence the pathological process of KOA through multiple pathways. The main mechanisms include: (1) suppressing appetite and reducing joint load; (2) improving insulin sensitivity and reducing systemic inflammation levels; (3) inhibiting inflammatory responses and regulating chondrocyte metabolism through activation of GLP-1 receptors; (4) promoting autophagy aiding in cellular homeostasis. These mechanisms may collectively contribute to slowing the progression of KOA. ADAMTS 4/5, A disintegrin and metalloproteinase with thrombospondin motifs 4/5; AGEs, Advanced glycation end products; Akt, Protein Kinase B; AMPK, AMP-activated Protein Kinase; ATP, Adenosine triphosphate; CHOP, C/EBP homologous protein; ER, Endoplasmic reticulum; GLP-1 RAs, Glucagon-like peptide-1 receptor agonists; IL, Interleukin; MAPK, Mitogen-activated protein kinase; MCP, Monocyte chemoattractant protein; MMP 1/3/13, Matrix metalloproteinase 1/3/13; MMP, Mitochondrial membrane potential; mTOR, Mammalian Target of Rapamycin; NF-κB, Nuclear Factor Kappa-B; NO, Nitric oxide; PGE2, Prostaglandin E2; RAGE, Receptor for advanced glycation end products; ROS, Reactive oxygen species; TNF-α, Tumor necrosis factor alpha.

The existing studies suggest that GLP-1 RAs protect against KOA through anti-inflammatory, anti-apoptotic, autophagy regulation, macrophage polarization mechanisms, etc. GLP-1 RAs reduce blood glucose, decreasing AGEs and their binding to RAGE receptors, which inhibits NF-κB and MAPK pathways, reducing pro-inflammatory factors like NO, PGE2, IL-1β, TNF-α, MCP-1, etc. They also lower 4-HNE, NOX-4, and ROS levels, reducing MMP-3/13 and ADAMTS-4/5 expression, thereby protecting type II collagen and aggrecan. Additionally, GLP-1 RAs inhibit ER stress via the PI3K/Akt pathway, decreasing CHOP, Caspase-3, and Bax while increasing Bcl-2, and restoring MMP and ATP levels in the mitochondrion, reducing ROS and apoptosis. They promote autophagy by lowering p62, increasing Beclin-1 and LC3-II, and activating AMPK while inhibiting mTOR, aiding in cellular homeostasis. GLP-1 RAs also shift macrophages from M1 to M2 type, altering the joint inflammatory microenvironment. These mechanisms collectively reduce inflammation, protect chondrocytes, regulate metabolism, and improve joint function, highlighting GLP-1 RAs’ therapeutic potential in KOA (Figure 3).

Preclinical studies demonstrate consistent dose- and time-dependent effects of GLP-1 RAs in osteoarthritis. Liraglutide (1–20 μg) (Meurot et al., 2022) and exenatide/lixisenatide (10–20 nM) (Li et al., 2019; Tong et al., 2019) showed dose-dependent efficacy, with higher doses providing stronger anti-inflammatory, anti-catabolic, and analgesic effects—20 μg liraglutide even outperformed dexamethasone. Temporally, liraglutide induced rapid (≤7 days) and sustained (≥28 days) benefits (Meurot et al., 2022), correlating with GLP-1 receptor/PKA/CREB activation, while untreated osteoarthritis saw progressive GLP-1 receptor decline (nadir at day 20) (Que et al., 2019). Exenatide/lixisenatide, though lacking multi-timepoint analyses, exerted acute NF-κB suppression (2–24 h) (Tong et al., 2019). These differences highlight the need for standardized protocols to reconcile dose- and time-response relationships in future research.

GLP-1 RAs such as liraglutide, exenatide, lixisenatide, dulaglutide, and geniposide share common chondroprotective mechanisms in osteoarthritis, including suppression of NF-κB (reducing IL-6, TNF-α, and COX-2), inhibition of matrix-degrading enzymes (MMP-3/13, ADAMTS-4/5), and attenuation OS (Meurot et al., 2022; Li et al., 2019; Tong et al., 2019). However, each agent exhibits distinct pathways: liraglutide activates PI3K/Akt and PKA/CREB while antagonizing RAGE (Que et al., 2019; Zhang et al., 2024); exenatide targets p38 MAPK pathway to suppress NF-κB and enhances glutathione (Tong et al., 2019); lixisenatide restores mitochondrial function (Li et al., 2019); and geniposide induces autophagy via AMPK/mTOR (Huang et al., 2023). These findings underscore the multifaceted therapeutic potential of GLP-1 RAs signaling in osteoarthritis, thereby providing a mechanistic framework to inform and refine future clinical trial design.

Furthermore, compared to traditional weight-loss interventions such as lifestyle modifications, bariatric surgery, or other pharmacotherapies (e.g., orlistat), GLP-1 RAs offer unique advantages, including combined glycemic control and weight reduction, as well as potential anti-inflammatory benefits specific to KOA. However, their higher cost and gastrointestinal side effects may limit accessibility and long-term adherence. While bariatric surgery demonstrates superior weight loss, GLP-1 RAs provide a less invasive option with broader metabolic effects. Future comparative studies are warranted to optimize patient stratification and treatment selection.

While GLP-1 RAs demonstrate therapeutic promise, their safety profile warrants nuanced evaluation. Gastrointestinal adverse events (e.g., nausea, vomiting) may stem from GLP-1 RAs-mediated delayed gastric emptying, particularly during dose escalation (Ard et al., 2021). Tumor-related concerns, though rare in trials, require vigilance given GLP-1 receptor expression in pancreatic and thyroid tissues (Waser et al., 2015; Yang et al., 2022). Long-term data reveal declining adherence (Lavu et al., 2024), possibly reflecting tolerability challenges. Importantly, the risk-benefit ratio favors obese/diabetic KOA patients, where metabolic benefits may outweigh risks, whereas non-metabolic populations necessitate caution pending further evidence.

This study has several limitations. First, given the substantial heterogeneity in experimental designs across preclinical studies (e.g., variations in animal models, dosing regimens, and *in vitro* experiments), we deliberately abstained from quality assessment of these investigations. Instead, our analysis focused on elucidating the consistent mechanistic pathways through which GLP-1 RAs may modulate KOA progression, as evidenced by convergent findings from *in vivo* and *in vitro* models. Second, quantitative pooling was not conducted due to the limited number of studies, diverse study types, and significant variability in outcome measures, which forced us to forego meta-analysis and may have led to the lack of persuasiveness of our findings. Third, our study solely investigated the effects of GLP-1 RAs on KOA; emerging evidence from mechanistic studies and observed risk reductions in osteoarthritis at other anatomical sites collectively point to a potential systemic therapeutic effect of these agents (Baser et al., 2024a; Baser et al., 2024b). Fourth, the mechanism of GLP-1 RAs in KOA is highly complex, and our proposed hypothesis requires further research and validation. Fifth, although GLP-1 RAs yielded statistically significant results in several outcomes—for example, intra-articular steroid injections fell by 0.087 per year—these changes may not translate into clinically meaningful benefit. Future research should therefore emphasize patient-centered endpoints to clarify the real-world value of GLP-1 RAs in KOA. Finally, while tirzepatide’s dual glucose-dependent insulinotropic polypeptide (GIP)/GLP-1 receptor agonism distinguishes it from selective GLP-1 RAs, we included it due to its shared GLP-1 receptor activation—the primary focus of our mechanistic review. Its inclusion aligns with our goal to explore the broader therapeutic potential of GLP-1 pathway modulation in KOA, though we acknowledge the need for future studies to dissect GIP-specific effects.

## Future directions

Based on the available studies, we found that many issues need to be addressed regarding GLP-1 RAs in improving KOA. First, there remains a paucity of robust evidence from multicenter, large-sample RCTs and real-world cohort studies. Second, variability in drug dosage, intervention duration, and follow-up time in clinical trials complicates the assessment of GLP-1 RAs’ efficacy, which has been shown to have dose- and time-dependent effects. Third, the clinical outcomes of different GLP-1 RAs vary widely, necessitating comparative studies. Fourth, comparative studies evaluating GLP-1 RAs against other antidiabetic medications (e.g., Metformin) in terms of clinical outcomes, mechanistic pathways, and safety profiles—especially for patients with concurrent diabetes and KOA—represent a critical area for future investigation. Fifth, higher adverse events, primarily gastrointestinal disorders and neoplasms, in the GLP-1 RAs group compared to controls, may hinder their widespread use. Sixth, while a potential mechanism of GLP-1 RAs in KOA has been proposed, further validation is needed due to its complexity. Seventh, a significant proportion of patients maintained or increased weight despite GLP-1 RAs treatment, which may suggest limited applicability to specific subgroups (e.g., age, sex, or diabetic status). Eighth, the efficacy of GLP-1 RAs in non-obese or non-diabetic KOA patients warrants further investigation. Ninth, we acknowledge the absence of fundamental studies involving semaglutide or tirzepatide in the current literature, and agree that future preclinical research on these agents would be valuable for elucidating their mechanisms in KOA. Finally, combining GLP-1 RAs with other interventions, such as dietary changes and physical activity, presents a promising research avenue.

## Conclusion

GLP-1 RAs held therapeutic potential for KOA patients with obesity or diabetes, but current evidence remained insufficient, warranting further high-quality RCTs and mechanistic studies to confirm their efficacy and safety.

## Glossary

4-HNE: 4-hydroxynonenal
ADAMTS-4/5: A disintegrin and metalloproteinase with thrombospondin motifs-4/5
AGEs: Advanced glycation end products
Akt: Protein Kinase B
AMPK: AMP-activated Protein Kinase
ATP: Adenosine triphosphate
BMI: Body mass index
CHOP: C/EBP homologous protein
COX-2: Cyclooxygenase 2
CREB: Cyclic adenosine monophosphate response element-binding protein
ECM: Extracellular matrix
ER: Endoplasmic reticulum
F: Female
GIP: Glucose-dependent insulinotropic polypeptide
GLP-1 RAs: Glucagon-like peptide-1 receptor agonists
GLP-1: Glucagon-like peptide-1
HbA1c: Hemoglobin A1c
ICOAP: Intermittent and Constant Osteoarthritis Pain
IL: Interleukin
K-L: Kellgren-Lawrence score
KOA: Knee osteoarthritis
KOOS: Knee Injury and Osteoarthritis Outcome Score
MAPK: Mitogen-activated protein kinase
MCP: Monocyte chemoattractant protein
MIA: Monosodium iodoacetate
MINORS: Methodological Index for Non-randomized Studies
MMP-1/3/13: Matrix metalloproteinase-1/3/13
MMP: Mitochondrial membrane potential
mTOR: Mammalian Target of Rapamycin
NF-κB: Nuclear Factor Kappa-B
NO: Nitric oxide
NOS: Newcastle-Ottawa scale
NOX-4: NADPH oxidase 4
NSAIDs: Non-steroidal anti-inflammatory drugs
OS: Oxidative stress
PGE2: Prostaglandin E2
PI3K: Phosphoinositide 3-Kinase
PKA: Protein Kinase A
PRISMA: Preferred Reporting Items for Systematic Reviews and Meta-Analyses
RAGE: Receptor for advanced glycation end products
RCT: Randomized controlled trial
ROS: Reactive oxygen species
SD: Standard deviation
SF-36: 36-Item Short Form Health Survey
T2D: Type 2 diabetes
TKA: Total knee arthroplasties
TNF-α: Tumor necrosis factor alpha
VAS: Visual analogue scale
WOMAC: Western Ontario and McMaster Universities Osteoarthritis Index.
