# Supplementary material for: Emerging therapeutic potential of glucagon-like Peptide-1 receptor agonists in knee osteoarthritis: a systematic review
Source: Front Pharmacol. 2025 Oct 13;16:1627691. doi: 10.3389/fphar.2025.1627691 (PMC12554640; doi:10.3389/fphar.2025.1627691)
Supplement: Supplementary file 1 [file DataSheet2.docx]

**1. Supplement1: Search strategy**

**2. Supplement2: Excluded articles at full-text assessment (n = 15)**

**1. Supplement1: Search strategy**

**Pubmed**

(("Glucagon-Like Peptide-1 Receptor Agonists"[Mesh]) OR ((((((((((((((((((((((((((((((((((((((((((((((((((((((((((((((((((((((((GLP 1[Title/Abstract]) OR (AC 2993[Title/Abstract])) OR (Adlyxin[Title/Abstract])) OR (albiglutide[Title/Abstract])) OR (AQVE-10010[Title/Abstract])) OR (AVE 0010[Title/Abstract])) OR (AVE 010[Title/Abstract])) OR (AVE0010[Title/Abstract])) OR (AVE-0010[Title/Abstract])) OR (AVE-010[Title/Abstract])) OR (beinaglutide[Title/Abstract])) OR (Bydureon[Title/Abstract])) OR (Byetta[Title/Abstract])) OR (DES-38-proline-exendine-4 (Heloderma suspectum)-(1-39)-peptidylpenta-l-lysyl-l-lysinamide[Title/Abstract])) OR (dulaglutide[Title/Abstract])) OR (Eperzan[Title/Abstract])) OR (Ex4 Peptide[Title/Abstract])) OR (Exenatide[Title/Abstract])) OR (Exendin 4[Title/Abstract])) OR (Exendin-4[Title/Abstract])) OR (GLP 1 Analogs[Title/Abstract])) OR (GLP 1 Receptor[Title/Abstract])) OR (GLP 1R Receptor[Title/Abstract])) OR (GLP-1[Title/Abstract])) OR (GLP-1-(7-36[Title/Abstract]))) OR (GLP1R Protein[Title/Abstract])) OR (GLP1R Receptor[Title/Abstract])) OR (GLP-1R Receptor[Title/Abstract])) OR (GLP-I (7-36[Title/Abstract]))) OR (Glucagon Like Peptide 1[Title/Abstract])) OR (Glucagon-Like Peptide 1[Title/Abstract])) OR (glucagon-like peptide 1 (7-36[Title/Abstract]))) OR (glucagon-like peptide I (7-36[Title/Abstract]))) OR (Glucagon-Like Peptide-1[Title/Abstract])) OR (Glucagon-Like Peptide-1 Receptor[Title/Abstract])) OR (Incretin Mimetics[Title/Abstract])) OR (ITCA 650[Title/Abstract])) OR (Liraglutide[Title/Abstract])) OR (lixisenatide[Title/Abstract])) OR (loxenatide[Title/Abstract])) OR (LY 2189265[Title/Abstract])) OR (LY-2189265[Title/Abstract])) OR (LY3298176[Title/Abstract])) OR (Lyxumia[Title/Abstract])) OR (Mashidutide[Title/Abstract])) OR (NN 2211[Title/Abstract])) OR (NN2211[Title/Abstract])) OR (NN-2211[Title/Abstract])) OR (Ozempic[Title/Abstract])) OR (Peptide, Ex4[Title/Abstract])) OR (Peptide-1 Receptor, Glucagon-Like[Title/Abstract])) OR (PEX168[Title/Abstract])) OR (polyethylene glycol loxenatide[Title/Abstract])) OR (proglucagon (78-107[Title/Abstract]))) OR (Protein, GLP1R[Title/Abstract])) OR (Receptor, GLP-1[Title/Abstract])) OR (Receptor, GLP1R[Title/Abstract])) OR (Receptor, GLP-1R[Title/Abstract])) OR (Receptor, Glucagon-Like Peptide-1[Title/Abstract])) OR (rGLP-1 protein[Title/Abstract])) OR (rybelsus[Title/Abstract])) OR (Saxenda[Title/Abstract])) OR (Semaglutide[Title/Abstract])) OR (Tanzeum[Title/Abstract])) OR (tirzepatide[Title/Abstract])) OR (Trulicity[Title/Abstract])) OR (Victoza[Title/Abstract])) OR (Wegovy[Title/Abstract])) OR (zepbound[Title/Abstract])) OR (ZP 10[Title/Abstract])) OR (ZP-10[Title/Abstract])) OR (ZP10A peptide[Title/Abstract]))) AND (("Osteoarthritis, Knee"[Mesh]) OR (((((((((((((((((KOA[Title/Abstract]) OR (Knee Osteoarthritis[Title/Abstract])) OR (Knee Osteoarthritides[Title/Abstract])) OR (knee OA[Title/Abstract])) OR (OA of the knee[Title/Abstract])) OR (degeneration of the knee[Title/Abstract])) OR (osteoarthritis of knee joint[Title/Abstract])) OR (Osteoarthritis Of Knees[Title/Abstract])) OR (Osteoarthritis of Knee[Title/Abstract])) OR (Osteoarthritis of the Knee[Title/Abstract])) OR (osteoarthritis[Title/Abstract])) OR (knee joint osseous arthritis[Title/Abstract])) OR (knee joint[Title/Abstract])) OR (knee arthritis[Title/Abstract])) OR (knee arthralgia[Title/Abstract])) OR (gonarthrosis[Title/Abstract])) OR (Knee*[Title/Abstract])))

**Search results: 79 items**

**Embase**

| **ID** | **Search** | **Results** |
| --- | --- | --- |
| #1 | osteoarthritis, knee':ab,ti | 988 |
| #2 | degeneration of the knee':ab,ti OR 'gonarthrosis':ab,ti OR 'knee arthralgia':ab,ti OR 'knee arthritis':ab,ti OR 'knee joint':ab,ti OR 'knee joint osseous arthritis':ab,ti OR 'knee oa':ab,ti OR 'knee osteoarthritides':ab,ti OR 'knee osteoarthritis':ab,ti OR 'koa':ab,ti OR 'oa of the knee':ab,ti OR 'osteoarthritis':ab,ti OR 'osteoarthritis of knee':ab,ti OR 'osteoarthritis of knee joint':ab,ti OR 'osteoarthritis of knees':ab,ti OR 'osteoarthritis of the knee':ab,ti OR 'knee*':ab,ti | 329894 |
| #3 | #1 OR #2 | 329894 |
| #4 | glucagon-like peptide-1 receptor':ab,ti | 8040 |
| #5 | glp 1':ab,ti OR 'glp 1 analogs':ab,ti OR 'glp 1 receptor':ab,ti OR 'glp 1r receptor':ab,ti OR 'glp-1':ab,ti OR 'glp-1 analogs':ab,ti OR 'glp-1 receptor':ab,ti OR 'glp1r protein':ab,ti OR 'glp1r receptor':ab,ti OR 'glp-1r receptor':ab,ti OR 'glucagon like peptide 1':ab,ti OR 'glucagon-like peptide 1':ab,ti OR 'glucagon-like peptide-1':ab,ti OR 'incretin mimetics':ab,ti OR 'peptide-1 receptor, glucagon-like':ab,ti OR 'protein, glp1r':ab,ti OR 'receptor, glp-1':ab,ti OR 'receptor, glp1r':ab,ti OR 'receptor, glp-1r':ab,ti OR 'receptor, glucagon-like peptide-1':ab,ti OR 'semaglutide':ab,ti OR 'ozempic':ab,ti OR 'rybelsus':ab,ti OR 'wegovy':ab,ti OR 'liraglutide':ab,ti OR 'nn 2211':ab,ti OR 'nn-2211':ab,ti OR 'nn2211':ab,ti OR 'victoza':ab,ti OR 'saxenda':ab,ti OR 'tirzepatide':ab,ti OR 'ly3298176':ab,ti OR 'zepbound':ab,ti OR 'rglp-1 protein':ab,ti OR 'tanzeum':ab,ti OR 'eperzan':ab,ti OR 'albiglutide':ab,ti OR 'exenatide':ab,ti OR 'ac 2993':ab,ti OR 'ac 2993 lar':ab,ti OR 'byetta':ab,ti OR 'bydureon':ab,ti OR 'itca 650':ab,ti OR 'exendin-4':ab,ti OR 'ex4 peptide':ab,ti OR 'peptide, ex4':ab,ti OR 'exendin 4':ab,ti OR 'glucagon-like peptide 1 (7-36)':ab,ti OR 'glp-1-(7-36)':ab,ti OR 'beinaglutide':ab,ti OR 'glp-i (7-36)':ab,ti OR 'glucagon-like peptide i (7-36)':ab,ti OR 'proglucagon (78-107)':ab,ti OR 'dulaglutide':ab,ti OR 'ly 2189265':ab,ti OR 'ly2189265':ab,ti OR 'ly-2189265':ab,ti OR 'trulicity':ab,ti OR 'polyethylene glycol loxenatide':ab,ti OR 'pex168':ab,ti OR 'lixisenatide':ab,ti OR 'des-38-proline-exendine-4 (heloderma suspectum)-(1-39)-peptidylpenta-l-lysyl-l-lysinamide':ab,ti OR 'ave 0010':ab,ti OR 'ave-0010':ab,ti OR 'ave0010':ab,ti OR 'aqve-10010':ab,ti OR 'lyxumia':ab,ti OR 'adlyxin':ab,ti OR 'ave 010':ab,ti OR 'ave-010':ab,ti OR 'zp10a peptide':ab,ti OR 'zp 10':ab,ti OR 'zp-10':ab,ti OR 'loxenatide':ab,ti OR 'mashidutide':ab,ti | 43286 |
| #6 | #4 or #5 | 43286 |
| #7 | #3 AND #6 | 92 |

**Search results: 92 items**

**Cochrane library**

| **ID** | **Search** | **Results** |
| --- | --- | --- |
| #1 | (Osteoarthritis, Knee):ti,ab,kw | 18618 |
| #2 | (degeneration of the knee):ti,ab,kw OR (gonarthrosis):ti,ab,kw OR (knee arthralgia):ti,ab,kw OR (knee arthritis):ti,ab,kw OR (knee joint):ti,ab,kw OR (knee joint osseous arthritis):ti,ab,kw OR (knee OA):ti,ab,kw OR (Knee Osteoarthritides):ti,ab,kw OR (Knee Osteoarthritis):ti,ab,kw OR (KOA):ti,ab,kw OR (OA of the knee):ti,ab,kw OR (osteoarthritis):ti,ab,kw OR (Osteoarthritis of Knee):ti,ab,kw OR (osteoarthritis of knee joint):ti,ab,kw OR (Osteoarthritis Of Knees):ti,ab,kw OR (Osteoarthritis of the Knee):ti,ab,kw OR (Knee*):ti,ab,kw | 51623 |
| #3 | #1 or #2 | 51623 |
| #4 | (Glucagon-Like Peptide-1 Receptor):ti,ab,kw | 1982 |
| #5 | (GLP 1):ti,ab,kw OR (GLP 1 Analogs):ti,ab,kw OR (GLP 1 Receptor):ti,ab,kw OR (GLP 1R Receptor):ti,ab,kw OR (GLP-1):ti,ab,kw OR (GLP-1 Analogs):ti,ab,kw OR (GLP-1 Receptor):ti,ab,kw OR (GLP1R Protein):ti,ab,kw OR (GLP1R Receptor):ti,ab,kw OR (GLP-1R Receptor):ti,ab,kw OR (Glucagon Like Peptide 1):ti,ab,kw OR (Glucagon-Like Peptide 1):ti,ab,kw OR (Glucagon-Like Peptide-1):ti,ab,kw OR (Incretin Mimetics):ti,ab,kw OR (Peptide-1 Receptor, Glucagon-Like):ti,ab,kw OR (Protein, GLP1R):ti,ab,kw OR (Receptor, GLP-1):ti,ab,kw OR (Receptor, GLP1R):ti,ab,kw OR (Receptor, GLP-1R):ti,ab,kw OR (Receptor, Glucagon-Like Peptide-1):ti,ab,kw OR (Semaglutide):ti,ab,kw OR (Ozempic):ti,ab,kw OR (rybelsus):ti,ab,kw OR (Wegovy):ti,ab,kw OR (Liraglutide):ti,ab,kw OR (NN 2211):ti,ab,kw OR (NN-2211):ti,ab,kw OR (NN2211):ti,ab,kw OR (Victoza):ti,ab,kw OR (Saxenda):ti,ab,kw OR (tirzepatide):ti,ab,kw OR (LY3298176):ti,ab,kw OR (zepbound):ti,ab,kw OR (rGLP-1 protein):ti,ab,kw OR (Tanzeum):ti,ab,kw OR (Eperzan):ti,ab,kw OR (albiglutide):ti,ab,kw OR (Exenatide):ti,ab,kw OR (AC 2993):ti,ab,kw OR (AC 2993 LAR):ti,ab,kw OR (Byetta):ti,ab,kw OR (Bydureon):ti,ab,kw OR (ITCA 650):ti,ab,kw OR (Exendin-4):ti,ab,kw OR (Ex4 Peptide):ti,ab,kw OR (Peptide, Ex4):ti,ab,kw OR (Exendin 4):ti,ab,kw OR (beinaglutide):ti,ab,kw OR (dulaglutide):ti,ab,kw OR (LY 2189265):ti,ab,kw OR (LY2189265):ti,ab,kw OR (LY-2189265):ti,ab,kw OR (Trulicity):ti,ab,kw OR (polyethylene glycol loxenatide):ti,ab,kw OR (PEX168):ti,ab,kw OR (lixisenatide):ti,ab,kw OR (AVE 0010):ti,ab,kw OR (AVE-0010):ti,ab,kw OR (AVE0010):ti,ab,kw OR (AQVE-10010):ti,ab,kw OR (Lyxumia):ti,ab,kw OR (Adlyxin):ti,ab,kw OR (AVE 010):ti,ab,kw OR (AVE-010):ti,ab,kw OR (ZP10A peptide):ti,ab,kw OR (ZP 10):ti,ab,kw OR (ZP-10):ti,ab,kw OR (loxenatide):ti,ab,kw OR (Mashidutide):ti,ab,kw | 10367 |
| #6 | #4 or #5 | 10367 |
| #7 | #3 and #6 | 43 |

**Search results: 43 items**

**Web of Science**

| **Set** | **Results** | **Search history** |
| --- | --- | --- |
| # 3 | 442 | #1 AND #2 |
| # 2 | 73,613 | TS=(Glucagon-Like Peptide-1 Receptor or GLP 1 or GLP 1 Analogs or GLP 1 Receptor or GLP 1R Receptor or GLP-1 or GLP-1 Analogs or GLP-1 Receptor or GLP1R Protein or GLP1R Receptor or GLP-1R Receptor or Glucagon Like Peptide 1 or Glucagon-Like Peptide 1 or Glucagon-Like Peptide-1 or Incretin Mimetics or Peptide-1 Receptor, Glucagon-Like or Protein, GLP1R or Receptor, GLP-1 or Receptor, GLP1R or Receptor, GLP-1R or Receptor, Glucagon-Like Peptide-1 or Semaglutide or olympic or rubellus or webovy or Liraglutide or NN 2211 or NN-2211 or nn2291 or victora or saxena or tirzepatide or ly329186 or zepound or rGLP-1 protein or tanzeem or ejerzan or albiglutide or Exenatide or AC 2903 or AC 2903 LAR or Byetta or Bydureon or itch 650 or Exendin-4 or Ex4 Peptide or Peptide, Ex4 or Exendin 4 or glucagon-like peptide 1 (7-36) or GLP-1-(7-36) or benaglutide or GLP-I (7-36) or glucagon-like peptide I (7-36) or proglucagon (78-107) or dulaglutide or LY 2180265 or LY2189265 or LY-2180265 or tropicity or polyethylene glycol losenatide or pex16p or lixisenatide or DES-38-proline-exendine-4 (helioderma suspected)-(1-39)-peptidylpenta-l-lysyl-l-lysinamide or AVE 0010 or AVE-0010 or ave001 or AQVE-10010 or ligumia or adlyxine or AVE 010 or AVE-010 or ze10a peptide or ZP 10 or ZP-10 or losenatide or Mashidutide) and Preprint Citation Index (Exclude – Database) |
| # 1 | 422,958 | TS=(Osteoarthritis, Knee or degeneration of the knee or gonarthrosis or knee arthralgia or knee arthritis or knee joint or knee joint osseous arthritis or knee OA or Knee Osteoarthritides or Knee Osteoarthritis or KOA or OA of the knee or osteoarthritis or Osteoarthritis of Knee or osteoarthritis of knee joint or Osteoarthritis Of Knees or Osteoarthritis of the Knee or Knee*) and Preprint Citation Index (Exclude – Database) |

**Search results: 442 items**

**2. Supplement2: Excluded articles at full-text assessment (n = 15)**

| **No.** | **Author and year** | **Reasons of exclusion** |
| --- | --- | --- |
| 1 | Ambrosio et al., 2014 | A case report |
| 2 | Baser et al., 2024a | OA risk involves other sites |
| 3 | Baser et al., 2024b | OA risk involves other sites |
| 4 | Buddhiraju et al., 2024 | Effects of GLP-1 receptor agonists on perioperative period of joint replacement surgery. |
| 5 | Elsabbagh et al., 2024 | Effects of GLP-1 receptor agonists on postoperative complications after shoulder replacement surgery. |
| 6 | Gudbergsen et al., 2019 | Protocol |
| 7 | Halabitska et al., 2024 | Review |
| 8 | Heckmann et al., 2024 | Review |
| 9 | Heo et al., 2024 | Effects of GLP-1 receptor agonists on postoperative complications of knee arthroplasty. |
| 10 | Jamal et al., 2024 | Review |
| 11 | Lawand et al., 2024 | Effects of GLP-1 receptor agonists on postoperative complications after shoulder replacement surgery. |
| 12 | Magruder et al., 2023 | Effects of GLP-1 receptor agonists on postoperative complications of knee arthroplasty. |
| 13 | Mayfield et al., 2024 | Review |
| 14 | Wang et al., 2019 | Letter |
| 15 | Wang et al., 2020 | Grape seed procyanidins ameliorates osteoarthritis the DPP4-Sirt1 pathway |

**References**

1. Ambrosio ML, Monami M, Sati L, Marchionni N, Di Bari M, Mannucci E. GLP-1 receptor agonist-induced polyarthritis: a case report. *Acta Diabetol*. 2014;51(4):673-674. doi:10.1007/S00592-013-0525-3
2. Baser O, Rodchenko K, Vivier E, Baser I, Lu Y, Mohamed M. The impact of approved anti-obesity medications on osteoarthritis. *Expert Opin Pharmacother*. 2024;25(11):1565-1573. doi:10.1080/14656566.2024.2391524
3. Baser O, Isenman L, Baser S, Samayoa G. Impact of semaglutide on osteoarthritis risk in patients with obesity: A retrospective cohort study. *Obes Sci Pract*. 2024;10(3):e762. doi:https://doi.org/10.1002/osp4.762
4. Buddhiraju A, Kagabo W, Khanuja HS, Oni JK, Nikkel LE, Hegde V. Decreased Risk of Readmission and Complications With Preoperative GLP-1 Analog Use in Patients Undergoing Primary Total Joint Arthroplasty. *J Arthroplasty*. 2024;39(12). doi:10.1016/J.ARTH.2024.05.079
5. Elsabbagh Z, Haft M, Murali S, Best M, McFarland EG, Srikumaran U. Does use of glucagon-like peptide-1 agonists increase perioperative complications in patients undergoing shoulder arthroplasty? *J Shoulder Elbow Surg*. Published online 2024. doi:10.1016/J.JSE.2024.07.045
6. Gudbergsen H, Henriksen M, Wæhrens EE, et al. Effect of liraglutide on body weight and pain in patients with overweight and knee osteoarthritis: protocol for a randomised, double-blind, placebo-controlled, parallel-group, single-centre trial. *BMJ Open*. 2019;9(5). doi:10.1136/BMJOPEN-2018-024065
7. Halabitska I, Babinets L, Oksenych V, Kamyshnyi O. Diabetes and Osteoarthritis: Exploring the Interactions and Therapeutic Implications of Insulin, Metformin, and GLP-1-Based Interventions. *Biomedicines*. 2024;12(8). doi:10.3390/BIOMEDICINES12081630
8. Heckmann ND, Palmer R, Mayfield CK, Gucev G, Lieberman JR, Hong K. Glucagon-Like Peptide Receptor-1 Agonists Used for Medically-Supervised Weight Loss in Patients With Hip and Knee Osteoarthritis: Critical Considerations for the Arthroplasty Surgeon. *Arthroplast Today*. 2024;27. doi:10.1016/J.ARTD.2024.101327
9. Heo KY, Goel RK, Fuqua A, et al. Glucagon-Like Peptide-1 Receptor Agonist Use is Not Associated With Increased Complications After Total Knee Arthroplasty in Patients With Type-2 Diabetes. *Arthroplast Today*. 2024;30. doi:10.1016/J.ARTD.2024.101506
10. Jamal N, Hollabaugh W, Scott L, Takkouche S. Unravelling the ties that bind: The intersection of obesity, osteoarthritis, and inflammatory pathways with emphasis on glucagon-like peptide-1 agonists. *Clin Obes*. 2025;15(1). doi:10.1111/COB.12700
11. Lawand JJ, Tansey PJ, Ghali A, et al. Glucagon-like peptide-1 receptor agonist use is associated with increased risk of perioperative complication and readmission following shoulder arthroplasty. *J Shoulder Elbow Surg*. Published online 2024. doi:10.1016/J.JSE.2024.09.012
12. Magruder ML, Yao VJH, Rodriguez AN, Ng MK, Sasson V, Erez O. Does Semaglutide Use Decrease Complications and Costs Following Total Knee Arthroplasty? *J Arthroplasty*. 2023;38(11):2311-2315.e1. doi:10.1016/J.ARTH.2023.05.071
13. Mayfield CK, Mont MA, Lieberman JR, Heckmann ND. Medical Weight Optimization for Arthroplasty Patients: A Primer of Emerging Therapies for the Joint Arthroplasty Surgeon. *J Arthroplasty*. 2024;39(1):38-43. doi:10.1016/J.ARTH.2023.07.017
14. Wang L, Feng L, Zhang J. Liraglutide exhibits anti-inflammatory activity through the activation of the PKA/CREB pathway. *J Inflamm (Lond)*. 2019;16(1). doi:10.1186/S12950-019-0225-Z
15. Wang K, Chen X, Chen Y, Sheng S, Huang Z. Grape seed procyanidins suppress the apoptosis and senescence of chondrocytes and ameliorates osteoarthritis via the DPP4-Sirt1 pathway. *Food Funct*. 2020;11(12):10493-10505. doi:10.1039/D0FO01377C
